# Measuring Positive Mental Health and Depression in Africa: A Variable-Based and Person-Centred Analysis of the Dual-Continua Model

**DOI:** 10.3389/fpsyg.2022.885278

**Published:** 2022-06-20

**Authors:** Itumeleng P. Khumalo, Richard Appiah, Angelina Wilson Fadiji

**Affiliations:** ^1^Department of Psychology, University of Johannesburg, Johannesburg, South Africa; ^2^Department of Occupational Therapy, College of Health Sciences, University of Ghana, Accra, Ghana; ^3^Center for African Studies, Harvard University, Cambridge, MA, United States; ^4^University of Pretoria, Pretoria, South Africa; ^5^North-West University, Potchefstroom, South Africa

**Keywords:** Africa, depression, latent class analysis, measurement, mental health continuum

## Abstract

The dual-continua model of mental health provides a contemporary framework for conceptualising and operationalising mental health. According to this model, mental health is distinct from but related to mental illness, and not the opposite or merely the absence of psychopathology symptoms. To examine the validity of the dual-continua model, previous studies have either applied variable-based analysis such as confirmatory factor analysis (CFA), or used predetermined cut-off points for subgroup division. The present study extends this contribution by subjecting data from an African sample to both CFA and latent class analysis (LCA) to test the dual-continua model in Africa. We applied CFA separately for the Mental Health Continuum—Short Form (MHC-SF) and Patient Health Questionnaire—9 (PHQ-9); and LCA on combined item responses. College students (*N* = 892; average age = 22.74, *SD* = 4.92; female = 58%) from Ghana (*n* = 309), Kenya (*n* = 262), Mozambique (*n* = 232), and South Africa (*n* = 89) completed the MHC-SF and PHQ-9. With minor modifications to the measurement models, the CFA results of this study confirm the three-factor structure of the MHC-SF, and a unidimensional solution for the PHQ-9. LCA results show the presence of three distinct latent classes: languishing with moderate endorsement of depressive symptoms (25.9%), flourishing with low endorsement of depressive symptoms (63.7%), and moderate mental health with high endorsement of depressive symptoms (10.4%). These findings further contribute to affirming the evidence for the dual-continua model of mental health, with implications for the assessment of mental health, to inform policy, practise, and future research in community and clinical settings in Africa.

## Introduction

Despite the emphasis placed on well-being in the description of (mental) health by the World Health Organization (1948, 2004), mental health researchers and practitioners have continued to conceptualise and measure mental health as the absence of (mental) illness (Ryff and Singer, 1998; Westerhof and Keyes, 2010; Deacon, 2013). Mental health ought to be conceptualised as a distinct but correlated construct with mental illness (Keyes, 2002, 2005, 2013). Keyes (Keyes and Lopez, 2002; Keyes, 2005, 2007, 2014) and others (e.g., Rose, 1981; Seligman, 2002; Huppert, 2005) have observed that although a focus on psychopathology is important, the exclusive weightage on deficits may have adverse implications for public mental health. To the extent that the absence or amelioration of symptoms of mental disorders does not indicate gains in positive mental health (Keyes and Lopez, 2002), an exclusive focus on diagnosing and treating mental disorders is unhelpful in preventing mental disorders and promoting public mental health, particularly in sub-Saharan Africa where the users of mental healthcare suffer stigma (Spittel et al., 2019; Appiah, 2022).

Responding to the problem of conceptualising and operationalising mental health in a comprehensive fashion, Keyes (2002, 2005, 2007) proposed the positive mental health model as a mental health continuum (MHC), ranging between languishing (incomplete) and flourishing (complete) mental health. Mental illness is represented by a separate continuum ranging from no and/or mild symptoms to severe symptoms of mental illnesses such as depression and anxiety (Westerhof and Keyes, 2010). The two constructs are distinct from each other and negatively correlated (Keyes, 2005, 2014; Westerhof and Keyes, 2010). From the coexistence and interaction of these two continua, the dual-continua model was conceived. Thus, the dual-continua model allows for the classification of individuals and the emergence of subgroups with distinct mental health status (Suldo and Shaffer, 2008; Keyes, 2014; Magalhães and Calheiros, 2017; Basson and Rothmann, 2018).

**Figure 1:**
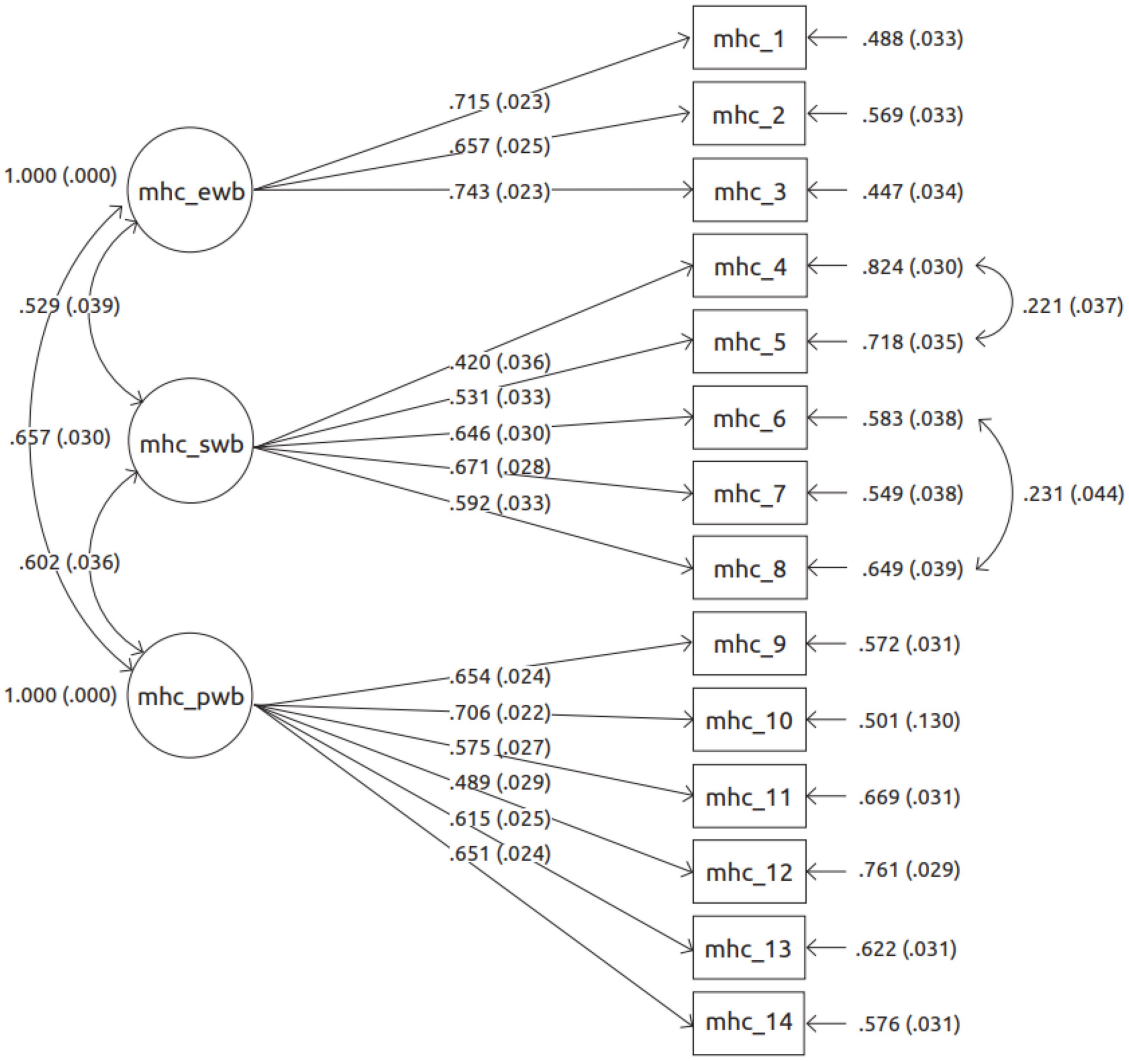
Best fitting measurement model for MHC-SF, showing standardised factor loadings.

Flourishing protects against psychopathology such as anxiety and depression (Schotanus-Dijkstra et al., 2017), as much as people can present with symptoms of mental health and mental illness simultaneously (Wong, 2011, 2013; Keyes et al., 2012). Compared to the single bipolar continuum model, the dual-continua model illustrates a more comprehensive classification of mental health (see Figure 1.1 of Keyes, 2013, p. 17 and Figure 1 of Iasiello and Van Agteren, 2020, p. 2). The dual-continua model takes into account the presence or absence of symptoms of mental disorders, together with the presence or absence of indicators of flourishing, which can co-occur to varying degrees in an individual. The four-quadrant model emanating from the mental illness and mental health continua has been used to group people *a priori* into four categories, namely (1) complete mental health, (2) symptomatic but content, (3) troubled, and (4) vulnerable (Suldo and Shaffer, 2008; Suldo et al., 2016; Magalhães and Calheiros, 2017; Iasiello and Van Agteren, 2020). Those with complete mental health are said to be well adjusted; and they are characterised by high subjective well-being levels and score low on psychopathology (Suldo et al., 2016). For students, belonging to this group is predictive of academic success (Suldo et al., 2016). The group categorised as symptomatic but content is also referred to as ambivalent and externally maladjusted. Although individuals in this category have elevated psychopathology levels, they also score average to high on mental health indices (Suldo et al., 2016). The troubled group is psychologically distressed, has poor mental health and experience symptoms of mental illness (Suldo et al., 2016). The vulnerable group has lower scores of mental health, yet does not make the clinical threshold for psychopathology (Suldo et al., 2016).

In a similar classification effort, using the same nomenclature, Keyes (2013) arrived at theoretically plausible categories based on three levels of mental health (I–III) and three of mental ill-health (IV–VI). Accordingly, an individual can be flourishing, meaning that they experience high levels of mental health (i.e., psychological, emotional, and social well-being) with low mental ill-health (I). Along the same continuum, an individual can be languishing (i.e., experience low mental health and low mental ill-health); (II), or experience moderate mental health, with low mental ill-health (III). Further, an individual can flourish and also experience mental ill-health (IV), experience both moderate mental health and mental ill-health (V), or languish with mental ill-health (VI). The model postulates that people can experience one of three mental health states (I–III), or experience any of these mental health states together with symptoms of mental ill-health (IV–VI) and different external correlates of functioning. It is, thus, possible for an individual to present with symptoms of mental ill-health such as depression, whilst simultaneously experiencing indicators of positive mental health. This configuration of mental health and mental illness on two possibly interacting continua makes for the dual-continua model of mental health (Keyes, 2005, 2007, 2013).

The dual-continua model is assessed by using measures of positive mental health indicators and symptoms of mental illness. The Mental Health Continuum-Short Form (MHC-SF: Keyes, 2005; Keyes et al., 2008) is a three-dimensional measure of (1) emotional well-being (EWB), (2) psychological well-being (PWB), and (3) social well-being (SWB). The EWB represents the presence of positive affect and satisfaction with life, as expressed by subjective well-being of Diener (2000) (hedonic). The PWB represents individuals’ intrapersonal and interpersonal functioning as indicated by the six dimensions of Ryff (1989, 2014), which are self-acceptance, purpose in life, autonomy, positive relations with others, environmental mastery, and personal growth. The SWB is an indication of how well an individual functions in society, is represented by social coherence, social acceptance, social actualisation, social contribution, and social integration.

The Patient Health Questionnaire serves as a measure of the presence and severity of depressive symptoms (Kroenke et al., 2001; Kroenke and Spitzer, 2002). The PHQ-9, which is often used to directly measure and screen for depressive symptoms in general and clinical groups, was chosen because the MHC positive mental health indicators were intended to parallel “the definition of depression in the DSM-IV, which includes both feelings of anhedonia (feeling sad or loss of interest and pleasure) and reported problems in functioning (such as problems in appetite, sleeping, or fatigue)” (Westerhof and Keyes, 2010, p. 112).

### The Present Study

Several empirical studies, mostly from Europe (e.g., Lamers et al., 2011a,b; Teismann et al., 2018) and America (e.g., Keyes, 2002, 2007; Keyes et al., 2010; Eklund et al., 2011; Renshaw et al., 2016), have examined the co-occurrence of positive mental health and mental illness, often by performing confirmatory factor analysis (CFA; Iasiello et al., 2020). For the majority of studies (e.g., Kim et al., 2014; Winzer et al., 2014; Magalhães and Calheiros, 2017), a two-factor oblique model fitted the data better than the single bipolar continuum and two orthogonal factors. This factor solution suggests that positive mental health and mental illness represent two distinct constructs, but which have a degree of overlap. A wealth of empirical research findings across population groups and contexts confirm two related factors to better fit the data (see Keyes et al., 2008; Renshaw and Cohen, 2014; Perugini et al., 2017).

A number of studies also set out to examine whether participants’ scores on measures of positive mental health (i.e., the MHC-SF) and mental illness (e.g., the PHQ-9) could lead to distinguishable subgroups in the dual-continua model (see Kelly et al., 2012; Lamers et al., 2015; Xiong et al., 2017). Relying on insights that well-being decreases the proclivity for psychopathology (Lamers et al., 2015); researchers have examined the dual-continua model by investigating the relations between mental health and symptoms of mental ill-health, drawing on scores of both measures of mental well-being and symptoms of psychopathology, often using latent class analysis (LCA). Of note, some studies have also investigated the validity of the dual-continua model by examining the extent to which indicators of positive mental health and mental illness correlate with external variables, such as self-efficacy (Schönfeld et al., 2016), coping strategies (Kinderman et al., 2015), and social support (Magalhães and Calheiros, 2017). These studies provided further evidence in support of the distinct yet correlated nature of mental health and mental illness.

Although some studies have examined the validity of the dual-continua model (e.g., Kelly et al., 2012; Bartels et al., 2013; Lyons et al., 2013; Suldo et al., 2016; Magalhães and Calheiros, 2017; Xiong et al., 2017; Yoo and Kahng, 2019), to our knowledge, no study has investigated the validity of the dual-continua model by applying CFA and LCA using scores on the MHC-SF and the PHQ-9, particularly in the sub-Saharan African context. Therefore, the present study investigated the operationalisation of the dual-continua model of mental health, through multiple statistical data analyses to investigate the validity of the dual-continua model using college students’ scores on the MHC-SF and PHQ-9. To achieve this goal, we first subjected the two measuring instruments, separately, to CFA, through which we could learn about their construct validity. Second, we subjected their items, together, to LCA, in order to identify how symptoms of mental health and mental illness show up in latent profiles across naturally occurring groups. These empirical research goals were informed by the theoretical position that mental health is distinct but related to mental illness (Keyes, 2002, 2005). The need to conduct this study with data from samples from multiple African countries was motivated by the knowledge that this group not only subscribes to greater collectivism but also conceptualises and experiences mental illness in different ways (e.g., Mosotho et al., 2008). These cultural variations are known to give effect to measurement properties (Chen, 2008), as well as how we understand both the operationalisation and the conceptualisation of models of mental health and mental illness.

## Materials and Methods

### Participants and Setting

Cross-sectional data from 892 participants (average age = 22.74, *SD* = 4.92; 58% women) from Ghana (*n* = 309), Kenya (*n* = 262), Mozambique (*n* = 232), and South Africa (*n* = 89) were included in the analyses for this study. All participants were university students at institutions from the four countries. Although these countries share different geographic and socioeconomic characteristics, they form part of the sub-Saharan African region of the world (see Figure 1), and have in common an African sociocultural orientation and a sociopolitical history of European colonialism (Møller and Roberts, 2017). Socio-biographical details of the participants, indicating their country, gender, age, and relationship status, are displayed in Table 1.

**Table 1:** Description of the sample by socio-biographical information per country.

Variable	Category	Ghana (309)		Kenya (262)		Moz (232)		SA (89)		Total	
		*F*	%	*F*	%	*F*	%	*F*	%	*F*	%
Age	≤18 years	54	17.48	8	3.05	20	8.62	12	13.48	92	10.3
	19 years	47	15.21	23	8.78	19	8.19	26	29.21	115	12.9
	20 years	55	17.80	38	14.50	15	6.47	14	15.73	122	13.7
	21 years	36	11.65	49	18.70	17	7.33	12	13.48	114	12.8
	22 years	35	11.33	33	12.60	14	6.03	8	9.00	90	10.1
	23 years	27	8.73	28	10.69	15	6.47	6	6.74	76	8.5
	24 years	12	3.88	12	4.58	18	7.76	1	1.12	43	4.8
	≥25 years	33	10.68	63	24.05	92	39.66	7	7.87	195	23.0
Sex	Male	102	33.01	149	56.87	85	36.63	31	34.83	367	41.4
	Female	207	66.99	112	42.74	144	62.07	56	62.92	519	58.2
Relationship	Single	287	92.88	204	77.86	146	62.93	85	95.51	722	80.9
	Cohabiting	79	25.6	19	7.25	55	23.71	1	1.12	79	8.9
	Married	63	20.39	33	12.60	17	7.33	0	0.00	63	7.1
	Divorced/widowed	8	2.59	3	1.15	5	2.16	0	0.00	8	0.9

### Measuring Instruments

#### Mental Health Continuum–Short Form

The MHC-SF is a 14-item self-report measure the items of which are responded to on a six-point Likert scale. It consists of three subscales measuring emotional well-being (EWB; three items), social well-being (SWB; five items), and psychological well-being (PWB; six items). Most studies (e.g., Lamers et al., 2011a,b; Diedericks and Rothmann, 2013; Schutte and Wissing, 2017; Appiah et al., 2022) have reported empirical evidence of the theoretically intended tripartite factor structure. Basson and Rothmann (2018) examined the MHC-SF latent profiles amongst a sample of South African students, using LCA, and found three groups. Researchers found poor omega reliability indices ranging between 0.41 and 0.57 for the MHC-SF subscales, but a high internal consistency for the general positive mental health factor (*ω* = 0.97) in a sample of rural Ghanaian adults (Appiah et al., 2022), partially confirming the underlying three-dimensional structure of mental health. Of note, Appiah et al. (2022) adopted interpretation of Perreira et al. (2018) that omega coefficients of *ω* > 0.50 represent satisfactory reliability for bifactor models. This finding from Ghanaian data attests to a possible unidimensional solution. In the present study, albeit with minor modification indices, the three subscales were retained and evidenced reliability. They attained the following omega reliability coefficients: 0.748 for EWB; 0.711 for SWB; and 0.786 for PWB (Keyes, 2006).

#### Patient Health Questionnaire-9

The PHQ-9 contains a collection of the nine symptoms of major depressive disorder as stipulated by the DSM. Often administered to both general and clinical samples, the PHQ-9 is used to screen for depression and monitor its severity and response to treatment. The PHQ-9 has shown excellent internal consistency, with Cronbach’s alpha values of between 0.86 and 0.89 in a group of primary care patients previously diagnosed with depression (Kroenke et al., 2001). In a sample of Nigerian university students, Adewuya et al. (2006) found reliability of 0.85. Amongst a sample of rural Ghanaian adults, Appiah et al. (2020a,b) also reported a reliability index of 0.78 for the PHQ-9 *Somatic symptoms* subscale, and 0.48 for the PHQ-9 *Non-somatic symptom* subscale. In the present study, we obtained omega reliability of 0.814 for the unidimensional PHQ-9 (Kroenke et al., 2001; Kroenke and Spitzer, 2002).

### Procedure and Ethical Aspects

At all the research sites, data were collected using a self-administered paper-and-pencil cross-sectional survey approach. Students were recruited through class announcements and pamphlets placed on their campuses. Ethical principles of informed consent and voluntary participation, in accordance with the Declaration of Helsinki (World Medical Association, 2013) and the South African Department of Health (DOH) (2014, 2015), were followed. The data for this study formed part of the data collected for a multi-country research project, entitled “*Measuring and exploring the contextual manifestation of well-being: A cross-cultural African study*,” which was granted ethical clearance by an accredited Research Ethics Committee based at the North-West University, South Africa (clearance number: NWU-HS-2015-0126). Ethical and institutional approval was obtained at each of the study sites prior to data collection.

### Data Analysis

Analyses were performed in Mplus (version 8.1; Muthén and Muthén, 1998-2017). After establishing well-fitting measurement models through CFA, we determined the reliability of the measuring instruments and descriptive statistics of the latent variables. Guidelines provided by Hu and Bentler (1999) and by Byrne (2012) were used to judge model fit: smaller and insignificant χ^2^, root mean square error of approximation (RMSEA), and a standardised root mean square residual (SRMR) of less than 0.06; a comparative fit index (CFI) of more than 0.95; a Tucker Lewis index (TLI) of more than 0.95; and a smaller Akaike information criterion (AIC) and a smaller Bayesian information criterion (BIC). Reliability was determined by estimating the omega reliability index. Unlike the popular Cronbach’s alpha, the omega index takes into account the effect of the variability of the contribution of each item to the latent variable through factor loadings (McNeish, 2017). We then applied LCA (Nylund et al., 2007; Rosato and Baer, 2012) to reveal distinct subgroups of students based on their responses to the MHC-SF and PHQ-9 items assessing symptoms of mental health and mental illness. To determine the optimal number of latent classes, the model fit indices of log-likelihood, AIC, BIC, sample size adjusted BIC, relative entropy, and probability values of Lo–Mendell–Rubin adjusted likelihood ratio test (LMR-LRT), Vuong-Lo–Mendell–Rubin likelihood ratio test (VLM-LRT) and parametric bootstrapped likelihood ratio test (PB-LRT) were considered. Models with an increasing number of latent classes were estimated and their fit compared across them until they yielded no further improvement, as specified by the k−1 guideline. Satisfactory fulfilment of this criterion was indicated by the non-significant probability value of LMR-LRT, VLM-LRT, and PB-LRT. Entropy values, which range between 0 and 1, were also considered with the view that high values (preferably higher than 0.80) indicate better classification, with the k−1 model being higher than the next model.

## Results

### MHC-SF Measurement Model Through CFA

As seen in Table 2, the best fitting MHC-SF model retained its tripartite factor structure of EWB, SWB, and PWB [*χ*^2^(72) = 336.613, *ρ* < 0.001; CFI = 924; TLI = 0.903; RMSEA = 0.064, *ρ* < 0.001, 90%CI [0.057 0.071]]. Nonetheless, this was after two pairs of items in the SWB subscale were allowed to have their error residuals covary. A modification index (MI) of 50.600 and an expected parameter change (EPC) of 0.541 informed the covariance between items 4 (*that you had something important to contribute to society*) and 5 [*that you belonged to a community (like a social group, or your neighbourhood)*]. The error residuals between item 6 (*that our society is becoming a better place for people like you*) and item 8 (*that the way our society works makes sense to you*) also covaried (MI = 21.79; EPC = 0.432). Figure 1 displays the factor loadings for the MHC-SF items. All the items attained significant loadings on their intended factors. For EWB, the following factor loadings were found: 0.743 for item 2; 0.715 for item 1; and 0.657 for item 2. For SWB, the factor loadings ranged between 0.420 (of item 4) and 0.671 (of item 7), whilst for PWB they ranged between 0.489 (item 12) and 0.706 (item 10).

**Table 2:** Model fit indices for the measurement models of the MHC-SF and PHQ-9 (*N* = 892).

Model	*χ* ^2^	*df*	*p*	RMSEA, *p* [90% CI]	CFI	TLI	SRMR	AIC	BIC
MHC-SF original	409.134	74	<0.001	0.071, <0.001 [0.065 0.078]	0.903	0.881	0.055	39,979	40,195
MHC-SF with 1 modification	357.470	73	<0.001	0.066, <0.001 [0.059 0.073]	0.918	0.898	0.057	39,930	40,150
MHC-SF with 2 modifications	336.613	72	<0.001	0.064, <0.001 [0.057 0.071]	0.924	0.903	0.050	39,911	40,136
PHQ-9 original	217.928	27	<0.001	0.089, <0.001 [0.078 0.100]	0.903	0.870	0.046	20,060	20,189
PHQ-9 with 1 modification	148.547	26	<0.001	0.073, 0.001 [0.062 0.084]	0.938	0.913	0.038	19,992	20,127

**χ*^2^, chi-square; df, degrees of freedom; *p*, probability estimate; CFI, comparative fit index; TLI, Tucker-Lewis index; RMSEA, root mean square error of approximation; SRMR, standardised root mean square residual; AIC, Akaike information criterion; BIC, Bayesian information criterion; MHC-SF, Mental Health Continuum Short-Form; and PHQ-9, Patient Health Questionnaire–9*.

### PHQ-9 Measurement Model Through CFA

**Figure 2:**
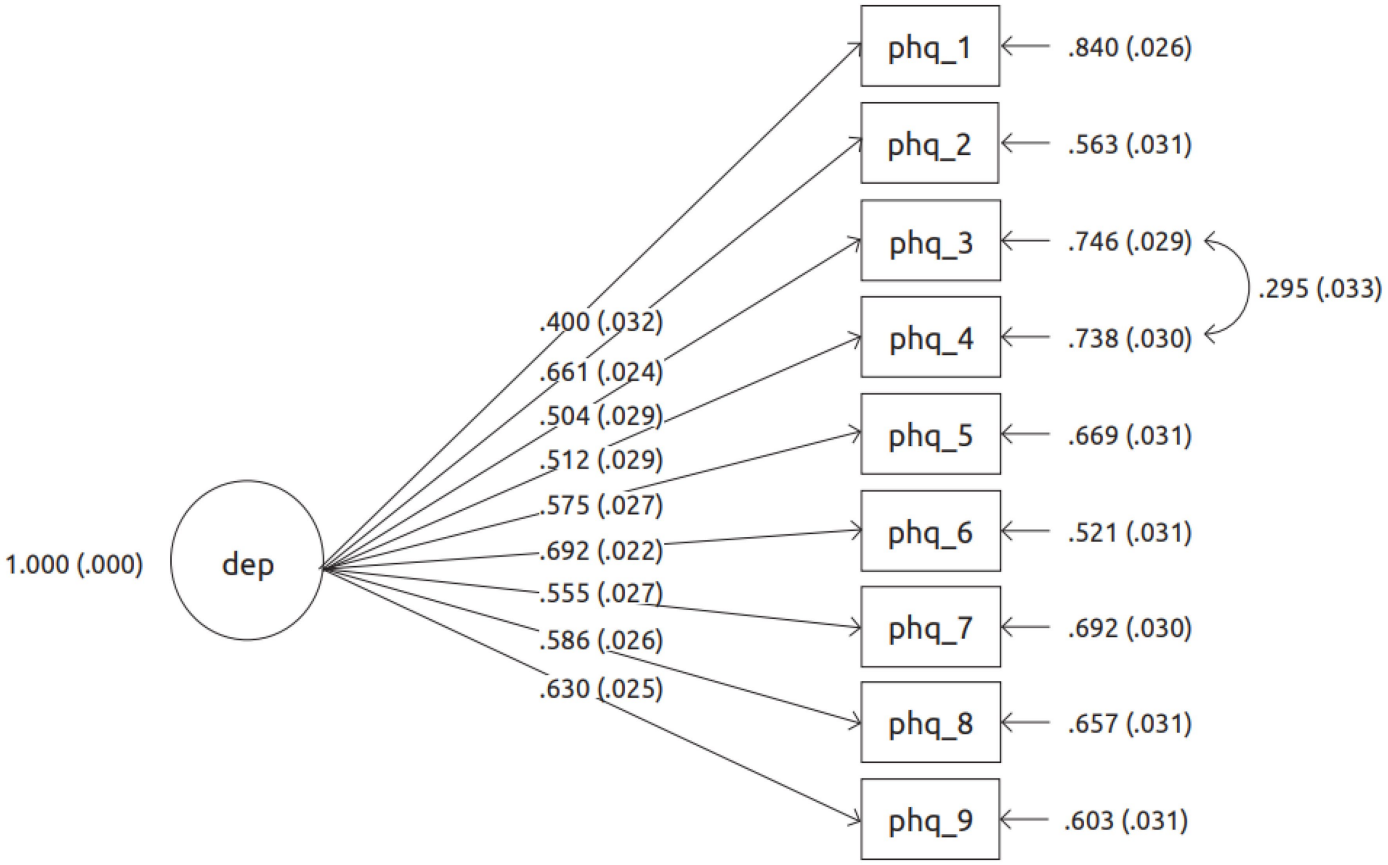
Best fitting measurement model for PHQ-9, showing standardised factor loadings.

As seen in Table 2, the unidimensional model of the PHQ-9, with a residual error covariance between items 3 (*Sleep disturbance*) and 4 (*Fatigued*; MI = 70.056; EPC = 0.221) demonstrated best fit [*χ*^2^(26) = 148.547, *ρ* < 0.001; CFI = 0.938; TLI = 0.913; RMSEA = 0.073, *ρ* = 0.001, 90%CI [0.062 0.084]]. This modification index application showed a significant improvement in the fit of the model as compared to the original one. The PHQ-9 items factor loadings, as displayed in Figure 2, ranged between 0.400 (item 1: *Anhedonia*) and 0.692 (item 6: *Guilt/worthlessness*).

### Descriptive Statistics for the MHC-SF Model

Descriptive statistics for the MHC-SF are reported in Table 3. The MHC-SF items are scored on a six-point Likert scale, ranging from 0 (*minimum*) to 5 (*maximum*). We deemed all the scores above 4 to be relatively high, and these were for MHC-SF item 2 [*interest in life*] (4.175), MHC-SF item 9 [*liking most parts of one’s personality*] (4.039), and MHC-SF item 14 [*life having a sense of direction or meaning*] (4.234). The three relatively low scores (i.e., below 3) were MHC-SF item 6 [*society becoming a better place*] (2.578), MHC-SF item 7 [*people are good*] (2.590), and MHC-SF item 8 [*making sense of how society works*] (2.271). All the skewness values were within the acceptable range of between −1.738 (MHC-SF item 14), and +0.247 (MHC-SF item 8). On the other hand, kurtosis values ranged between −1.321 (MHC-SF item 6) and +2.483 (MHC-SF item 14).

**Table 3:** Item-level descriptive statistics of the MHC-SF Items, based on the best fitting model, for the whole sample (*n* = 892).

Variable	Mean	Variance	Skewness	Kurtosis	Range		Percentiles				Median
					Min	Max	20%	60%	40%	80%	
MHC 1	3.696	1.113	−1.256	1.638	0.00	5.00	3.00	4.00	4.00	4.00	4.00
MHC 2	4.175	1.218	−1.450	1.600	0.00	5.00	3.00	5.00	4.00	5.00	5.00
MHC 3	3.382	1.839	−0.963	0.257	0.00	5.00	2.00	4.00	3.00	4.00	4.00
MHC 4	3.128	2.600	−0.525	−0.936	0.00	5.00	1.00	4.00	3.00	5.00	4.00
MHC 5	3.444	2.727	−0.823	−0.586	0.00	5.00	2.00	4.00	4.00	5.00	4.00
MHC 6	2.578	3.026	−0.129	−1.321	0.00	5.00	1.00	3.00	2.00	4.00	3.00
MHC 7	2.590	2.327	−0.195	−1.097	0.00	5.00	1.00	3.00	2.00	4.00	3.00
MHC 8	2.271	2.821	0.247	−0.546	0.00	5.00	1.00	3.00	2.00	4.00	2.00
MHC 9	4.039	1.325	−1.391	1.588	0.00	6.00	3.00	5.00	4.00	5.00	4.00
MHC 10	3.893	1.326	−1.223	1.196	0.00	5.00	3.00	4.00	4.00	5.00	4.00
MHC 11	3.516	2.039	−0.949	−0.037	0.00	6.00	2.00	4.00	4.00	5.00	4.00
MHC 12	3.914	1.671	−1.238	0.786	0.00	6.00	3.00	5.00	4.00	5.00	4.00
MHC 13	3.698	1.749	−1.049	0.470	0.00	6.00	3.00	4.00	4.00	5.00	4.00
MHC 14	4.234	1.361	−1.738	2.483	0.00	5.00	4.00	5.00	4.00	5.00	5.00

*MHC, Mental Health Continuum; Min, Minimum; and Max, Maximum*.

### Descriptive Statistics for the PHQ-9 Model

Descriptive statistics for the PHQ-9 are reported in Table 4. Within a minimum and maximum range of between 0 and 4, the lowest item mean score for the PHQ-9 was 0.323 (PHQ-9 item 9: *suicide ideation*) and the highest was 1.221 (PHQ-9 item 1: *anhedonia*). Skewness values ranged between +0.343 (PHQ-9 item 1) and +2.381 (PHQ-9 item 9). Kurtosis values ranged between −1.139 (PHQ-9 item 1) and +4.656 (PHQ-9 item 9).

**Table 4:** Item-level descriptive statistics of the PHQ-9 items, based on the best fitting model, for the whole sample (*n* = 892).

Variable	Mean	Variance	Skewness	Kurtosis	Range		Percentiles				Median
					Min	Max	20%	60%	40%	80%	
PHQ-9 1	1.221	1.134	0.343	−1.139	0.00	3.00	0.00	1.00	1.00	2.00	1.00
PHQ-9 2	0.824	0.858	0.863	−0.221	0.00	4.00	0.00	1.00	0.00	2.00	1.00
PHQ-9 3	0.850	1.038	0.819	−0.652	0.00	3.00	0.00	1.00	0.00	2.00	0.00
PHQ-9 4	1.099	0.986	0.503	−0.823	0.00	3.00	0.00	1.00	1.00	2.00	1.00
PHQ-9 5	0.794	1.027	0.964	−0.380	0.00	3.00	0.00	1.00	0.00	2.00	0.00
PHQ-9 6	0.540	0.775	1.528	1.241	0.00	3.00	0.00	0.00	0.00	1.00	0.00
PHQ-9 7	0.807	0.952	0.922	−0.331	0.00	3.00	0.00	1.00	0.00	2.00	0.00
PHQ-9 8	0.550	0.756	1.425	0.910	0.00	3.00	0.00	0.00	0.00	1.00	0.00
PHQ-9 9	0.323	0.573	2.381	4.656	0.00	3.00	0.00	0.00	0.00	0.00	0.00

*PHQ-9, Patient Health Questionnaire; Min, Minimum; amd Max, Maximum*.

### Latent Class Analysis of MHC-SF and PHQ-9 Items Together

As displayed in Table 5, a three-class model (AIC = 61,225; BIC = 61,675; SSABIC = 61,377; Entropy = 914; LMR-LRT *ρ* = 0.0072; VLM-LRT *ρ* = 0.0069; and PB-LRT *ρ* < 0.001) fitted the data best as compared to its competing models (i.e., one-, two-, and four-class models). This three-class model is characterised by lower AIC, BIC, and SSABIC values and the highest Entropy value. The fourth model with four classes did not demonstrate improved fit indices, but instead presented non-significant LMR-LRT and VLM-LRT probability values. The classification predictions, as seen in Table 6, support the model fit results. They show 92.6, 97.1, and 98.3% of the probability of belonging for class 1, 2, and 3, respectively.

**Table 5:** Latent class solution model fit indices using the indicators of MHC-SF and PHQ-9 (*N* = 892).

Model	Log likelihood	AIC	BIC	SSA BIC	Entropy	LMR- LRT *ρ*	VLM- LRT *ρ*	PB-LRT *ρ*	Percentage			
									Class 1	Class 2	Class 3	Class 4
1 class	−32,411	64,914	65,134	64,988	.	.	.	.	100	.	.	.
2 classes	−31,149	62,439	62,774	62,552	0.890	0.0091	0.0089	<0.001	72.5	27.5	.	.
**3 classes**	**−30,518**	**61,225**	**61,675**	**61,377**	**0.914**	**0.0072**	**0.0069**	**<0.001**	**25.8**	**63.7**	**10.4**	.
4 classes	−30,195	60,626	61,191	60,817	0.906	0.3352	0.3328	<0.001	34.1	5.4	52.0	8.5

*AIC, Akaike information criterion; BIC, Bayesian information criterion; SSA BIC, sample size adjusted BIC; LMR-LRT, Lo–Mendell–Rubin adjusted likelihood ratio test; VLM-LRT, Vuong-Lo–Mendell–Rubin LRT; and PB-LRT, parametric bootstrapped LRT*.

*Bold values mean best fitting class solution*.

**Table 6:** Likelihood of belonging: classification probabilities of the most likely latent class membership (column) by latent class (row).

	Class 1 (%)	Class 2 (%)	Class 3 (%)
Class 1	**0.926**	0.073	0.001
Class 2	0.028	**0.971**	0.001
Class 3	0.017	0.000	**0.983**

*Bold values mean likelihood of belonging*.

**Figure 3:**
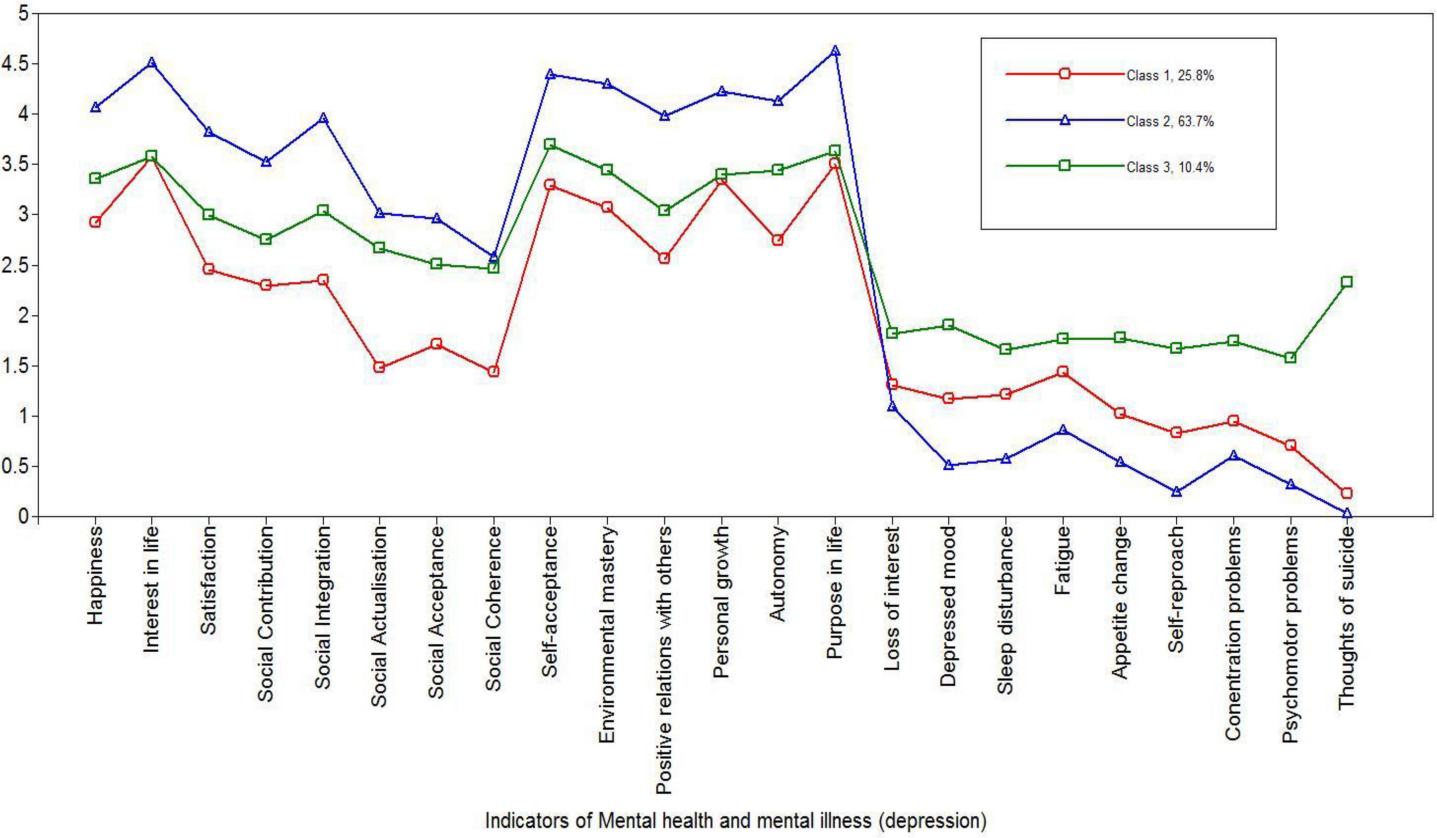
Latent classes profile.

Based on the latent class profiles (displayed in Figure 3), we labelled the three classes as follows: *Languishing* with moderate endorsement of depressive symptoms (25.9%), *Flourishing* with least endorsement of depressive symptoms (63.7%), and *Moderate mental health* with high endorsement of depressive symptoms (10.4%).

## Discussion

The purpose of the present study was to investigate psychometric support for the dual-continua model of mental health through conducting CFA and LCA on the responses to the MHC-SF and PHQ-9 by university students from four African countries. Our findings provide support for the dual-continua model as operationalised using MHC-SF and PHQ-9. In addition to the empirical support for the hypothesised model, there is also empirical evidence for the context-informed nuances of how the dual-continua model finds expression amongst students in Africa. These nuanced variations are found in the specific item residual error covariances, levels and distribution of mean scores, as well as homogeneous groupings of how mental health and illness indicators interact/coexist in the same individuals.

### Measuring Positive Mental Health Using the MHC-SF

Our data provided empirical support for the tripartite factor structure of the MHC-SF made up of emotional, psychological, and social well-being. The model yielded high reliabilities and significant factor loadings on the theoretically intended dimensions/target factors. Although some studies have found similar results in the past (e.g., Keyes et al., 2008; Khumalo et al., 2012), others have had different evidence also emerge (e.g., Santini et al., 2020; Lebares et al., 2021; Appiah et al., 2022). Santini et al. (2020) found the MHC-FS as a composite measure to be reliable, and its factor structure to be better accounted for by a bipolar solution, yet with low reliabilities for the subscales. Results of Santini et al. (2020) point to the possibility of a more salient unidimensional general well-being factor.

The three-factor model from the present data had two important modifications. These modifications were the covariance of error residuals between items 4 (*one has something important to contribute: social contribution*) and 5 (*one belonged to a community: social belonging*), and between 6 (*society becoming a better place: social actualisation*) and 8 (*the way society works makes sense: social coherence*) of the social well-being dimension. This finding dovetails with previous report and supposition from the sub-Saharan African context that people may see themselves to contribute (item 4) to communities to which they feel they belong (item 5), and that society would be seen to become better (item 6) when it is run/working well (item 8; Schutte and Wissing, 2017; Appiah et al., 2022). Similar to our finding, Santini et al. (2020) found the social well-being items, specifically item 6 (*society becoming a better place*) and item 4 (*one has something important to contribute*) to be problematic. A possible explanation for this finding is that participants perceived the evaluation of the improvement of society (item 6) and their contributions to society (item 4) as part of their overall positive mental health, but not as part of a clearly distinguished social well-being factor.

Appiah et al. (2022) surmised that individuals from highly collectivistic African societies could experience some levels of intrapsychic conflict and the psychological burden when tasked to self-evaluate themselves or their contributions to others or society. In the Ghanaian collectivistic cultural context, for instance, self-evaluations in the public space could be interpreted as a show of pride or boasting (Gyekye, 2010; Appiah, 2020). On the other hand, Santini et al. (2020) found that the *society becoming a better place* item was interpreted by participants as not tapping on individual character/well-being, but rather on how others run society; whilst the *one has something important to contribute* item had been found by the participants too vague and difficult to answer.

### Measuring Depression Using the PHQ-9

The data from the present study supported a unidimensional factor solution to describe depression. The unidimensionality of depression (Baas et al., 2011; Bhana et al., 2015; Galenkamp et al., 2017) is one of at least three positions held about this syndrome. Others have made a distinction between somatic and non-somatic symptoms (Kendel et al., 2010; Elhai et al., 2012; Forkmann et al., 2013; Beard et al., 2016; Appiah et al., 2020a,b; Tadi et al., 2022), whilst the differentiation across emotional, cognitive, and somatic symptoms is also not uncommon. However, the unity of depressive symptoms in this study was characterised by two important features, the first being the covariance in the error residuals of items 3 (sleep disturbance) and 4 (fatigue), and the second being the non-normal distribution of item 9 (suicide ideation), which was not only scored the lowest, but had a clustering of scores at the low values where they are extremely peaked. This information tells us of the overlap in manifestation of sleep disturbance and fatigue in this group, as well as their adamant denial of possibilities of suicide ideation and self-harm.

### Mental Health and Illness Show Up in Three Latent Classes

**Figure 4:**
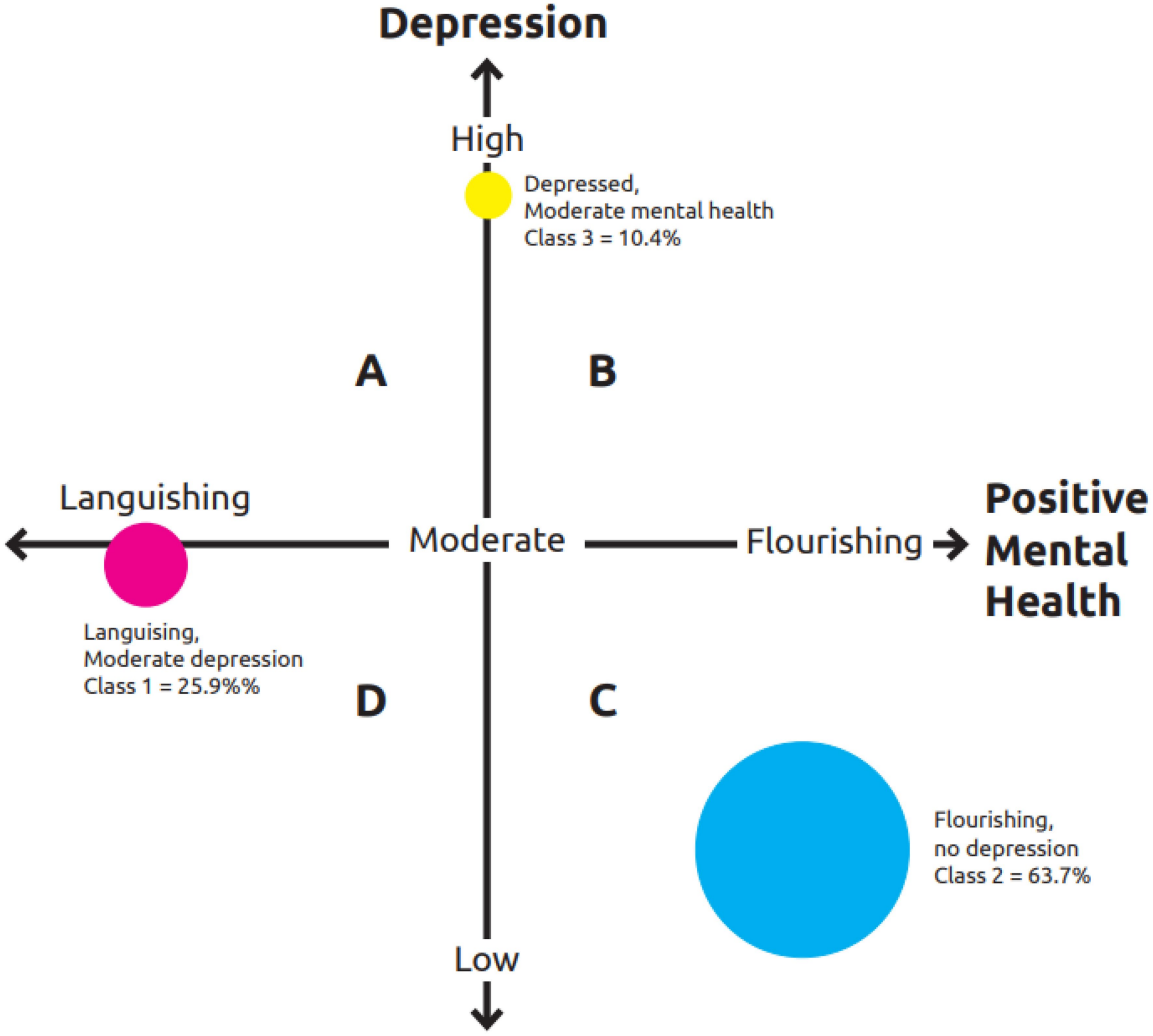
The three latent classes on a matrix of positive mental health and depression.

As displayed in Figure 4, LCA results of the combined 14 indicators of mental health and nine indicators of depression yielded three latent classes. The first class, comprising 25.9% of the sample, was labelled “*Languishing with moderate endorsement of depressive symptoms.*” The second class was made up of the majority of the sample (i.e., 63.7%), and was named “*Flourishing with least endorsement of depressive symptoms.*” The third and last class, consisting of 10.4% of the sample, was called “*Moderate mental health with high endorsement of depressive symptoms.*” With varying degrees of difference in approaches and choices of inclusion and exclusion, several previous studies have used clustering methods to explore the dual-continuum model (e.g., Basson and Rothmann, 2018). Analysis of Basson and Rothmann (2018) only comprised responses to the MHC-SF, and found three classes.

The conceptually postulated cluster model characterising mental health and mental illness indicators is an empirical data-supported four-quadrant matrix (Suldo and Shaffer, 2008; Keyes, 2013; Suldo et al., 2016; Magalhães and Calheiros, 2017; Iasiello and Van Agteren, 2020). These studies have described four groups, with which our study’s finding of three latent classes partially overlap. It is important to note that our homogeneous groups based on the endorsement of positive mental health and ill-health indicators were not determined *a priori*, but were naturally underlying the data and emerged through LCA. These three groups are interpreted in the context of the theoretical postulations and empirical finding of Keyes (2013) and Suldo and Shaffer (2008) and Suldo et al. (2016). The Languishing with moderate endorsement of depressive symptoms latent class is reminiscent of languishing with mental illness (VI) group of Keyes (2013). In our sample, it made up 25.9% of the participants. These are the individuals Suldo and colleagues would classify as troubled. The class we called Flourishing with least endorsement of depressive symptoms dominated the sample at 63.7%. This is a class similar to group I of Keyes (2013), which is characterised by flourishing and low mental illness. In Suldo’s classification, it is called complete mental health. Accordingly, 10.4% of our sample was moderately mentally healthy as much as they also presented with high levels of depression. This group resembles group V of Keyes (2013). When we consider Suldo’s classification, this group is neither vulnerable nor symptomatic but content. However, the group is at greater risk than the subclinical threshold which characterises Suldo’s vulnerable classification.

### Limitations and Recommendations

Notwithstanding the contribution made by this study, it is limited in that it included only a convenience sample of university students with a limited age range, not representative of the general population across a lifespan. To expand and enhance the inquiry into the dual-continua model and further make sense of our findings, a number of recommendations for future research are made. In addition to quantitative data analysis, future research could include a qualitative examination of participants’ understanding of the construct indicators included in the measurements (e.g., Santini et al., 2020). Qualitative data could also be used to explore the differences in the characteristics and experiences of individuals across the latent classes (e.g., Khumalo et al., 2022). Future correlational and group comparison studies could shed light on the external determinants and behavioural outcomes of the naturally occurring homogeneous groups (e.g., Magalhães and Calheiros, 2017). Studies with external criteria may demonstrate the causal/predictive relationships through longitudinal designs. As had been previously found (e.g., Joshanloo et al., 2013; Santini et al., 2020), further exploration of the internal consistency of the social well-being dimension is necessary.

### Conclusion and Implications

Although several studies have conducted CFA and LCA (often separately) to examine the dual-continua model across contexts, this study contributes to the existing body of knowledge by exploring the dual-continua model in the sub-Saharan African context. Our finding has implications for practise in a few unique ways. Empirical evidence of the dual-continuum model highlights the characteristics of the subclinical groups who could still be at high risk and could benefit from mental health promotion interventions (Vella-Brodrick, 2013; Appiah et al., 2020a,b). An adoption of the dual-continua model and research and practise scope could instigate efforts and strategies to identify those who have low well-being and low pathology, but are below the threshold mark for psychopathology (Magalhães and Calheiros, 2017; Appiah, 2022). Bhugra et al. (2022) places the advocacy responsibility for the prevention of mental illness and its stigma, and the promotion of mental health on the shoulders of practitioners. In the end, “the importance of measuring mental health in the same way as mental illness cannot be overstated” (Keyes, 2013, p. 16).

## Data Availability Statement

The raw data supporting the conclusions of this article will be made available by the authors, without undue reservation.

## Ethics Statement

The studies involving human participants were reviewed and approved by Research Ethics Committee of the North-West University, South Africa. The patients/participants provided their written informed consent to participate in this study.

## Author Contributions

IK, RA, and AW contributed equally to idea conceptualisation, data management, and manuscript writing. All authors contributed to the article and approved the submitted version.

## Funding

The study was supported by the South African National Research Foundation (NRF) through two grants: grant number (129386 to AW) and grant number (112092 to IK).

## Conflict of Interest

The authors declare that the research was conducted in the absence of any commercial or financial relationships that could be construed as a potential conflict of interest.

## Publisher’s Note

All claims expressed in this article are solely those of the authors and do not necessarily represent those of their affiliated organizations, or those of the publisher, the editors and the reviewers. Any product that may be evaluated in this article, or claim that may be made by its manufacturer, is not guaranteed or endorsed by the publisher.

## References

[ref1] Adewuya A. O. Ola B. A. Afolabi O. O. (2006). Validity of the patient health questionnaire (PHQ-9) as a screening tool for depression amongst Nigerian university students. J. Affect. Disord. 96, 89–93. doi: 10.1016/j.jad.2006.05.021, PMID: 16857265

[ref2] Appiah R. (2020). Community-based participatory research in rural African contexts: ethico-cultural considerations and lessons from Ghana. Public Health Rev. 41, 1–13. doi: 10.1186/s40985-020-00145-2, PMID: 33292760 PMC7694909

[ref3] Appiah R. (2022). A look back, a path forward: revisiting the mental health and well-being research and practice models and priorities in sub-Saharan Africa. New Ideas Psychol. 65:100931. doi: 10.1016/j.newideapsych.2022.100931

[ref4] Appiah R. Schutte L. Wilson Fadiji A. Wissing M. P. Cromhout A. (2020a). Factorial validity of the Twi versions of five measures of mental health and well-being in Ghana. PLoS One 15:e0236707. doi: 10.1371/journal.pone.0236707, PMID: 32780773 PMC7418998

[ref5] Appiah R. Wilson-Fadiji A. Schutte L. Wissing M. P. (2020b). Effects of a community-based multicomponent positive psychology intervention on mental health of rural adults in Ghana. Appl. Psychol. Health Well Being 12, 828–862. doi: 10.1111/aphw.12212, PMID: 32706933

[ref6] Appiah R. Wissing M. P. Wilson Fadiji A. Schutte L. (2022). “Factorial validity of the Twi version of the mental health continuum-short form and prevalence of mental health in a rural Ghanaian sample,” in Embracing Well-Being in Diverse African Contexts: Research Perspectives. Cross-Cultural Advancements in Positive Psychology. eds. Schutte L. Guse T. Wissing M. P. (Cham: Springer), 16.

[ref7] Baas K. D. Cramer A. O. J. Koeter M. W. J. Van de Lisdonk E. H. Van Weert H. C. Schene A. H. (2011). Measurement invariance with respect to ethnicity of the patient health questionnaire-9 (PHQ-9). J. Affect. Disord. 129, 229–235. doi: 10.1016/j.jad.2010.08.026, PMID: 20888647

[ref8] Bartels M. Cacioppo J. T. Van Beijsterveldt T. C. Boomsma D. I. (2013). Exploring the association between well-being and psychopathology in adolescents. Behav. Genet. 43, 177–190. doi: 10.1007/s10519-013-9589-7, PMID: 23471543 PMC3897864

[ref9] Basson M. J. Rothmann S. (2018). Flourishing: positive emotion regulation strategies of pharmacy students. Int. J. Pharm. Pract. 26, 458–464. doi: 10.1111/ijpp.12420, PMID: 29239045

[ref10] Beard C. Hsu K. J. Rifkin L. S. Busch A. B. Björgvinsson T. (2016). Validation of the PHQ-9 in a psychiatric sample. J. Affect. Disord. 193, 267–273. doi: 10.1016/j.jad.2015.12.075, PMID: 26774513

[ref11] Bhana A. Rathod S. D. Selohilwe O. Kathree T. Petersen I. (2015). The validity of the patient health questionnaire for screening depression in chronic care patients in primary health care in South Africa. BMC Psychiatry 15:118. doi: 10.1186/s12888-015-0503-0 26001915 PMC4446842

[ref12] Bhugra D. Tribe R. Poulter D. (2022). Social justice, health equity, and mental health. S. Afr. J. Psychol. 52, 3–10. doi: 10.1177/00812463211070921

[ref13] Byrne B. M. (2012). Structural equation modeling with mplus: basic concepts, applications, and programming. New York: Routledge.

[ref14] Chen F. F. (2008). What happens if we compare chopsticks with forks? The impact of making inappropriate comparisons in cross-cultural research. J. Pers. Soc. Psychol. 95, 1005–1018. doi: 10.1037/a0013193, PMID: 18954190

[ref15] Deacon B. J. (2013). The biomedical model of mental disorder: a critical analysis of its validity, utility, and effects on psychotherapy research. Clin. Psychol. Rev. 33, 846–861. doi: 10.1016/j.cpr.2012.09.007, PMID: 23664634

[ref16] Diedericks E. Rothmann S. (2013). Flourishing of information technology professionals: the role of work engagement and job satisfaction. J. Psychol. Afr. 23, 225–233. doi: 10.1080/14330237.2013.10820618

[ref17] Diener E. (2000). Subjective well-being: the science of happiness and a proposal for a national index. Am. Psychol. 55, 34–43. doi: 10.1037/0003-066X.55.1.34, PMID: 11392863

[ref18] Eklund K. Dowdy E. Jones C. Furlong M. (2011). Applicability of the dual-factor model of mental health for college students. J. Coll. Stud. Psychother. 25, 79–92. doi: 10.1080/87568225.2011.532677

[ref19] Elhai J. D. Contractor A. A. Tamburrino M. Fine T. H. Prescott M. R. Shirley E.. (2012). The factor structure of major depression symptoms: a test of four competing models using the patient health Questionnaire-9. Psychiatry Res. 199, 169–173. doi: 10.1016/j.psychres.2012.05.018, PMID: 22698261

[ref20] Forkmann T. Gauggel S. Spangenberg L. Brähler E. Glaesmer H. (2013). Dimensional assessment of depressive severity in the elderly general population: psychometric evaluation of the PHQ-9 using Rasch analysis. J. Affect. Disord. 148, 323–330. doi: 10.1016/j.jad.2012.12.019, PMID: 23411025

[ref21] Galenkamp H. Stronks K. Snijder M. B. Derks E. M. (2017). Measurement invariance testing of the PHQ-9 in a multi-ethnic population in Europe: the HELIUS study. BMC Psychiatry 17:349. doi: 10.1186/s12888-017-1506-9, PMID: 29065874 PMC5655879

[ref001] Gyekye K. (2010). “African ethics,” in Stanford Encyclopedia of Philosophy. ed. Zalta E.. Available at: https://plato.stanford.edu/entries/african-ethics

[ref22] Hu L. T. Bentler P. M. (1999). Cutoff criteria for fit indixes in covariance structure analysis: conventional criteria versus new alternatives. Struct. Equ. Model. 6, 1–55. doi: 10.1080/10705519909540118

[ref23] Huppert F. A. (2005). “Positive mental health in individuals and populations,” in The Science of Well-Being. eds. Huppert F. A. Baylis N. Keverne B. (New York: Oxford University Press), 307–340.

[ref24] Iasiello M. Van Agteren J. (2020). Mental health and/or mental illness: a scoping review of the evidence and implications of the dual-continua model of mental health. Evid. Base 1, 1–45. doi: 10.21307/eb-2020-001

[ref25] Iasiello M. Van Agteren J. Muir-Cochrane E. (2020). Mental health and/or mental illness: a scoping review of the evidence and implications of the dual-continua model of mental health. Evid. Base 1, 1–45. doi: 10.21307/eb-2020-001

[ref26] Joshanloo M. Wissing M. P. Khumalo I. P. Lamers S. M. A. (2013). Measurement invariance of the mental health continuum-short form (MHC-SF) across three cultural groups. J. Person. Individ. Differ. 55, 755–759. doi: 10.1016/j.paid.2013.06.002

[ref27] Kelly R. M. Hills K. J. Huebner E. Mcquillin S. D. (2012). The longitudinal stability and dynamics of group membership in the dual-factor model of mental health: psychosocial predictors of mental health. Can. J. Sch. Psychol. 27, 337–355. doi: 10.1177/0829573512458505

[ref28] Kendel F. Wirtz M. Dunkel A. Lehmkuhl E. Hetzer R. Regitz-Zagrosek V. (2010). Screening for depression: Rasch analysis of the dimensional structure of the PHQ-9 and the HADS-D. J. Affect. Disord. 122, 241–246. doi: 10.1016/j.jad.2009.07.004, PMID: 19665236

[ref30] Keyes C. L. M. (2002). The mental health continuum: from languishing to flourishing in life. J. Health Soc. Res. 43, 207–222. doi: 10.2307/3090197, PMID: 12096700

[ref31] Keyes C. L. M. (2005). Mental illness and/or mental health? Investigating axioms of the complete state model of health. J. Consult. Clin. Psychol. 73, 539–548. doi: 10.1037/0022-006X.73.3.539, PMID: 15982151

[ref32] Keyes C. L. M. (2006). Mental health in adolescence: is America’s youth flourishing? Am. J. Orthop. 76, 395–402. doi: 10.1037/0002-9432.76.3.395, PMID: 16981819

[ref33] Keyes C. L. (2007). Promoting and protecting mental health as flourishing: a complementary strategy for improving national mental health. Am. Psychol. 62, 95–108. doi: 10.1037/0003-066X.62.2.95, PMID: 17324035

[ref35] Keyes C. L. M. (2013). “Promoting and protecting positive mental health: early and often throughout the lifespan,” in Mental Well-Being: International Contributions to the Study of Positive Mental Health. ed. Keyes C. L. M. (Dordrecht: Springer), 3–28.

[ref36] Keyes C. L. M. (2014). “Mental health as a complete state: how the salutogenic perspective completes the picture,” in Bridging Occupational, Organizational and Public Health. eds. Bauer G. F. Hämmig O. (Dordrecht: Springer), 179–192.

[ref37] Keyes C. L. M. Dhingra S. S. Simoes E. J. (2010). Change in level of positive mental health as a predictor of future risk of mental illness. Am. J. Public Health 100, 2366–2371. doi: 10.2105/AJPH.2010.192245, PMID: 20966364 PMC2978199

[ref38] Keyes C. L. Eisenberg D. Perry G. S. Dube S. R. Kroenke K. Dhingra S. S. (2012). The relationship of level of positive mental health with current mental disorders in predicting suicidal behavior and academic impairment in college students. J. Am. Coll. Heal. 60, 126–133. doi: 10.1080/07448481.2011.608393, PMID: 22316409

[ref39] Keyes C. L. M. Lopez S. J. (2002). “Toward a science of mental health: positive directions in diagnosis and interventions,” in Handbook of Positive Psychology. eds. Snyder C. R. Lopez S. J. (New York: Oxford University Press), 45–59.

[ref40] Keyes C. L. Wissing M. Potgieter J. P. Temane M. Kruger A. Van Rooy S. (2008). Evaluation of the mental health continuum-short form (MHC-SF) in Setswana-speaking south Africans. Clin. Psychol. Psychother. 15, 181–192. doi: 10.1002/cpp.572, PMID: 19115439

[ref41] Khumalo I. P. De Klerk W. Fadiji A. W. (2022). “Nature and role of student hope and meaning in goal setting: implications for higher education in South Africa,” in Embracing Well-Being in Diverse African Contexts: Research Perspectives. Cross-Cultural Advancements in Positive Psychology. eds. Schutte L. Guse T. Wissing M. P. (Cham: Springer), 16.

[ref42] Khumalo I. P. Temane Q. M. Wissing M. P. (2012). Socio-demographic variables, general psychological well-being and the mental health continuum in an African context. Soc. Indic. Res. 105, 419–442. doi: 10.1007/s11205-010-9777-2

[ref43] Kim E. K. Furlong M. J. Dowdy E. Felix E. D. (2014). Exploring the relative contributions of the strength and distress components of dual-factor complete mental health screening. Can. J. Sch. Psychol. 29, 127–140. doi: 10.1177/0829573514529567

[ref44] Kinderman P. Tai S. Pontin E. Schwannauer M. Jarman I. Lisboa P. (2015). Causal and mediating factors for anxiety, depression and well-being. Br. J. Psychiatry 206, 456–460. doi: 10.1192/bjp.bp.114.147553, PMID: 25858180

[ref45] Kroenke K. Spitzer R. L. (2002). The PHQ-9: a new depression diagnostic and severity measure. Psychiatr. Ann. 32, 509–515. doi: 10.3928/0048-5713-20020901-06, PMID: 35168093

[ref46] Kroenke K. Spitzer R. L. Williams J. B. (2001). The PHQ-9: validity of a brief depression severity measure. J. Gen. Intern. Med. 16, 606–613. doi: 10.1046/j.1525-1497.2001.016009606.x, PMID: 11556941 PMC1495268

[ref47] Lamers S. A. Glas C. A. W. Westerhof G. J. Bohlmeijer E. T. (2011a). Longitudinal evaluation of the mental health continuum-short form (MHC-SF): measurement invariance across demographics, physical illness, and mental illness. Eur. J. Psychol. Assess. 28, 290–296. doi: 10.1027/1015-5759/a000109

[ref48] Lamers S. A. Westerhof G. F. Bohlmeijer E. T. ten Klooster P. M. Keyes C. L. M. (2011b). Evaluating the psychometric properties of the mental health continuum-short form (MHC-SF). J. Clin. Psychol. 67, 99–110. doi: 10.1002/jclp.20741, PMID: 20973032

[ref49] Lamers S. M. A. Westerhof G. J. Glas C. A. W. Bohlmeijer E. T. (2015). The bidirectional relation between positive mental health and psychopathology in a longitudinal representative panel study. J. Posit. Psychol. 10, 553–560. doi: 10.1080/17439760.2015.1015156

[ref50] Lebares C. C. Greenberg A. L. Shui A. Boscardin C. van der Schaaf M. (2021). Flourishing as a measure of global well-being in first year residents: a pilot longitudinal cohort study. J. Med. Educ. Curric. Dev. 8:23821205211020758. doi: 10.1177/23821205211020758, PMID: 34104793 PMC8170288

[ref51] Lyons M. D. Huebner E. Hills K. J. (2013). The dual-factor model of mental health: A short-term longitudinal study of school-related outcomes. Soc. Indic. Res. 114, 549–565. doi: 10.1007/s11205-012-0161-2

[ref53] Magalhães E. Calheiros M. M. (2017). A dual-factor model of mental health and social support: evidence with adolescents in residential care. Child Youth Serv. Rev. 70, 442–449. doi: 10.1016/j.childyouth.2017.06.041

[ref54] McNeish D. (2017). Thanks coefficient alpha, we’ll take it from here. Psychol. Methods 23, 412–433. doi: 10.1037/met0000144, PMID: 28557467

[ref55] Møller V. Roberts B. (2017). “New beginnings in an ancient region: well-being in sub-Saharan Africa,” in The Pursuit of Human Well-Being: The Untold Global History. International Handbooks of Quality-of-Life. eds. Estes R. J. Sirgy M. J. (Cham: Springer), 161–215.

[ref56] Mosotho N. L. Louw D. A. Calitz F. J. Esterhuyse K. G. (2008). Depression among Sesotho speakers in Mangaung. Afri. J. Psychiatry 11, 35–43. doi: 10.4314/ajpsy.v11i1.30253, PMID: 19582323

[ref57] Muthén L. K. Muthén B. O. (1998–2017). Mplus Statistical Analysis With Latent Variables: Users’ Guide. 8th *Edn.* Los Angeles, CA: Muthén & Muthén.

[ref58] Nylund K. L. Asparouhov T. Muthén B. O. (2007). Deciding on the number of classes in latent class analysis and growth mixture modeling: a Monte Carlo simulation study. Struct. Equ. Model. Multidiscip. J. 14, 535–569. doi: 10.1080/10705510701575396

[ref002] Perreira T. A. Morin A. J. S. Hebert M. Gillet N. Houle S. A. Berta W. (2018). The short form of the Workplace Affective Commitment Multidimensional Questionnaire (WACMQ-S): a bifactor-ESEM approach among healthcare professionals. J. Vocat. Behav. 106, 62–83. doi: 10.1016/j.jvb.2017.12.004, PMID: 28344677

[ref59] Perugini M. L. L. de la Iglesia G. Solano A. C. Keyes C. L. M. (2017). The mental health continuum–short form (MHC–SF) in the Argentinean context: confirmatory factor analysis and measurement invariance. Eur. J. Psychol. 13:93. doi: 10.5964/ejop.v13i1.1163, PMID: 28344677 PMC5342313

[ref61] Renshaw T. L. Cohen A. S. (2014). Life satisfaction as a distinguishing indicator of college student functioning: further validation of the two-continua model of mental health. Soc. Indic. Res. 117, 319–334. doi: 10.1007/s11205-013-0342-7

[ref62] Renshaw T. L. Eklund K. R. Bolognino S. J. Adodo I. (2016). Bidimensional emotional health in college students: a comparison of categorical and continuous analytic approaches. J. Psychopathol. Behav. Assess. 38, 681–694. doi: 10.1007/s10862-016-9558-6

[ref63] Rosato N. S. Baer J. C. (2012). Latent class analysis: a method for capturing heterogeneity. Soc. Work. Res. 36, 61–69. doi: 10.1093/swr/svs006

[ref64] Rose G. (1981). Strategy of prevention: lessons from cardiovascular disease. Br. Med. J. 282, 1847–1851. doi: 10.1136/bmj.282.6279.1847 6786649 PMC1506445

[ref65] Ryff C. D. (1989). Happiness is everything, or is it? Explorations on the meaning of psychological well-being. J. Pers. Soc. Psychol. 57, 1069–1081. doi: 10.1037/0022-3514.57.6.1069, PMID: 35500357

[ref66] Ryff C. D. (2014). Psychological well-being revisited: advances in the science and practice of eudaimonia. Psychother. Psychosom. 83, 10–28. doi: 10.1159/000353263, PMID: 24281296 PMC4241300

[ref67] Ryff C. D. Singer B. (1998). The contours of positive human health. Psychol. Inq. 9, 1–28. doi: 10.1207/s15327965pli0901_1

[ref68] Santini Z. I. Torres-Sahli M. Hinrichsen C. Meilstrup C. Madsen K. R. Rayce S. B.. (2020). Measuring positive mental health and flourishing in Denmark: validation of the mental health continuum-short form (MHC-SF) and cross-cultural comparison across three countries. Health Qual. Life Outcomes 18:297. doi: 10.1186/s12955-020-01546-2, PMID: 32887631 PMC7650216

[ref69] Schönfeld P. Brailovskaia J. Bieda A. Zhang X. C. Margraf J. (2016). The effects of daily stress on positive and negative mental health: mediation through self-efficacy. Int. J. Clin. Health Psychol. 16, 1–10. doi: 10.1016/j.ijchp.2015.08.005, PMID: 30487845 PMC6225043

[ref70] Schotanus-Dijkstra M. Drossaert C. Pieterse M. E. Boon B. Walburg J. A. Bohlmeijer E. T. (2017). An early intervention to promote well-being and flourishing and reduce anxiety and depression. A randomized controlled trial. Internet Interv. 9, 15–24. doi: 10.1016/j.invent.2017.04.002, PMID: 30135833 PMC6096189

[ref71] Schutte L. Wissing M. P. (2017). Clarifying the factor structure of the mental health continuum short form in three languages: a bifactor exploratory structural equation modeling approach. Soc. Mental Health 7, 142–158. doi: 10.1177/2156869317707793

[ref72] Seligman M. E. P. (2002). “Positive psychology, positive prevention, and positive therapy,” in Handbook of Positive Psychology. eds. Snyder C. R. Lopez S. J. (New York: Oxford University Press), 3–9.

[ref73] South African Department of Health (DOH) (2014). National Health Act (Act No. 61 of 2003) Regulations relating to research with human participants. Government Gazette No. R. 719. Government Printing Works.

[ref74] South African Department of Health (DOH) (2015). Ethics in health research: Principles, structures and processes. Available at: https://www.health.gov.za/wp-content/uploads/2021/10/nhrec-docs_38000199NationalRegulationCV01.pdf

[ref75] Spittel S. Maier A. Kraus E. (2019). Awareness challenges of mental health disorder and dementia facing stigmatisation and discrimination: a systematic literature review from sub-Sahara Africa. J. Glob. Health 9:020419. doi: 10.7189/jogh.09.020419, PMID: 31656607 PMC6790232

[ref76] Suldo S. M. Shaffer E. J. (2008). Looking beyond psychopathology: the dual-factor model of mental health in youth. Sch. Psychol. Rev. 37, 52–68. doi: 10.1080/02796015.2008.12087908

[ref77] Suldo S. M. Thalji-Raitano A. Kiefer S. M. Ferron J. M. (2016). Conceptualizing high school students’ mental health through a dual-factor model. Sch. Psychol. Rev. 45, 434–457. doi: 10.17105/SPR45-4.434-457

[ref78] Tadi N. F. Pillay K. Ejoke U. P. Khumalo I. P. (2022). Sex differences in depression and anxiety symptoms: measurement invariance, prevalence, and symptom heterogeneity among university students in South Africa. Front. Psychol. 13:873292. doi: 10.3389/fpsyg.2022.873292 35712197 PMC9195165

[ref79] Teismann T. Brailovskaia J. Siegmann P. Nyhuis P. Wolter M. Willutzki U. (2018). Dual factor model of mental health: co-occurrence of positive mental health and suicide ideation in inpatients and outpatients. Psychiatry Res. 260, 343–345. doi: 10.1016/j.psychres.2017.11.085, PMID: 29232575

[ref80] Vella-Brodrick D. A. (2013). “Positive psychology interventions: research evidence, practical utility, and future steps,” in Mental Well-Being: International Contributions to the Study of Positive Mental Health. ed. Keyes C. L. M. (Dordrecht: Springer), 331–353.

[ref82] Westerhof G. J. Keyes C. L. (2010). Mental illness and mental health: The two continua model across the lifespan. J. Adult Dev. 17, 110–119. doi: 10.1007/s10804-009-9082-y 20502508 PMC2866965

[ref83] Winzer R. Lindblad F. Sorjonen K. Lindberg L. (2014). Positive versus negative mental health in emerging adulthood: a national cross-sectional survey. BMC Public Health 14, 1–10. doi: 10.1186/1471-2458-14-1238, PMID: 25438621 PMC4265536

[ref84] Wong P. T. (2011). Positive psychology 2.0: towards a balanced interactive model of the good life. Can. Psychol. 52:69. doi: 10.1037/a0022511

[ref85] Wong P. T. (2013). Positive psychology. Encyclop. Cross Cult. Psychol. 3, 1021–1027. doi: 10.1002/9781118339893.wbeccp426

[ref86] World Health Organization (1948). World health organization constitution. Basic Doc. 1:22.

[ref87] World Health Organization (2004). Promoting Mental Health: Concepts, Emerging Evidence, Practice—Summary report. Author.

[ref88] World Medical Association (2013). World Medical Association Declaration of Helsinki Ethical Principles for Medical Research Involving Human Subjects. 10.1001/jama.2013.281053 24141714

[ref89] Xiong J. Qin Y. Gao M. Hai M. (2017). Longitudinal study of a dual-factor model of mental health in Chinese youth. Sch. Psychol. Int. 38, 287–303. doi: 10.1177/0143034317689970

[ref90] Yoo C. Kahng S. K. (2019). Two-dimensional mental health and related predictors among adolescents in Korea. Asian Soc. Work Policy Rev. 13, 66–77. doi: 10.1111/aswp.12157

